# Replicative and Air Pollution-Induced Senescence: Telomere Dysfunction, Mitochondrial Stress, and Modulatory Effects of Astragaloside IV in Endothelial and Vascular Smooth Muscle Cells

**DOI:** 10.1007/s12012-026-10173-0

**Published:** 2026-09-11

**Authors:** Paola Canale, Francesca Forini, Giuseppina Nicolini, Lisa Alibrandi, Francesca Scebba, Antonella Mercuri, Stefano Turchi, Irene Marinaro, Jonica Campolo, Maria Grazia Andreassi

**Affiliations:** 1 CNR Institute of Clinical Physiology, Pisa and Milan, Italy; 2 Health Science Interdisciplinary Centre, Sant’Anna School of Advanced Studies, Pisa, Italy

**Keywords:** Vascular senescence, Telomere dysfunction, Mitochondrial stress, Vascular cells, Fine dust, Astragaloside IV

## Abstract

Telomere dysfunction and mitochondrial impairment are central drivers of vascular cell senescence and atherosclerosis, but their molecular interplay—especially under environmental stress—remains poorly defined. Fine particulate matter (PM) is an important contributor to vascular aging, but effective strategies to mitigate its detrimental effects are still lacking. This study investigated telomeric and mitochondrial alterations in human umbilical vein endothelial cells (HUVECs) and human coronary artery  smooth muscle cells (hVSMCs) during replicative senescence (RS) and stress-induced premature senescence (SIPS), and evaluated the potential protective effects of Astragaloside IV (AS-IV), a natural compound with telomere and mitochondria-protective properties, against PM-induced vascular senescence. RS was induced by serial passaging and categorized as young (passages 6–7), intermediate (10–11), and old (17–20). SIPS was induced in early-passage cells by 48-h exposure to fine dust (FD), a PM_10_-like certified reference material representative of urban traffic emissions, at concentrations of 50 and 100 μg/mL. For the intervention experiments, cells were exposed to FD at 100 μg/mL for 48 h, followed by AS-IV (50 μM) for 24 h. Both HUVECs and hVSMCs showed hallmark features of RS, including SA-β-gal positivity, telomere shortening, activation of p16^INK4a^/p21^Cip1^, and mitochondrial dysfunction. FD-induced SIPS predominantly affected HUVECs, leading to premature senescence, telomere shortening, TERRA upregulation, and acute mitochondrial impairment, whereas hVSMCs showed relative resistance despite telomere shortening. AS-IV partially attenuated FD-induced telomeric and mitochondrial damage in HUVECs but had only limited effects in hVSMCs, underscoring the endothelial susceptibility to FD. These findings demonstrate that vascular senescence is governed by cell type-specific telomere-mitochondria interactions that are differentially modulated by FD. The selective protective effects of AS-IV in endothelial cells support the development of telomere-targeted therapeutic strategies to mitigate pollution-induced vascular aging.

**Graphical Abstract:**

**Figure:**
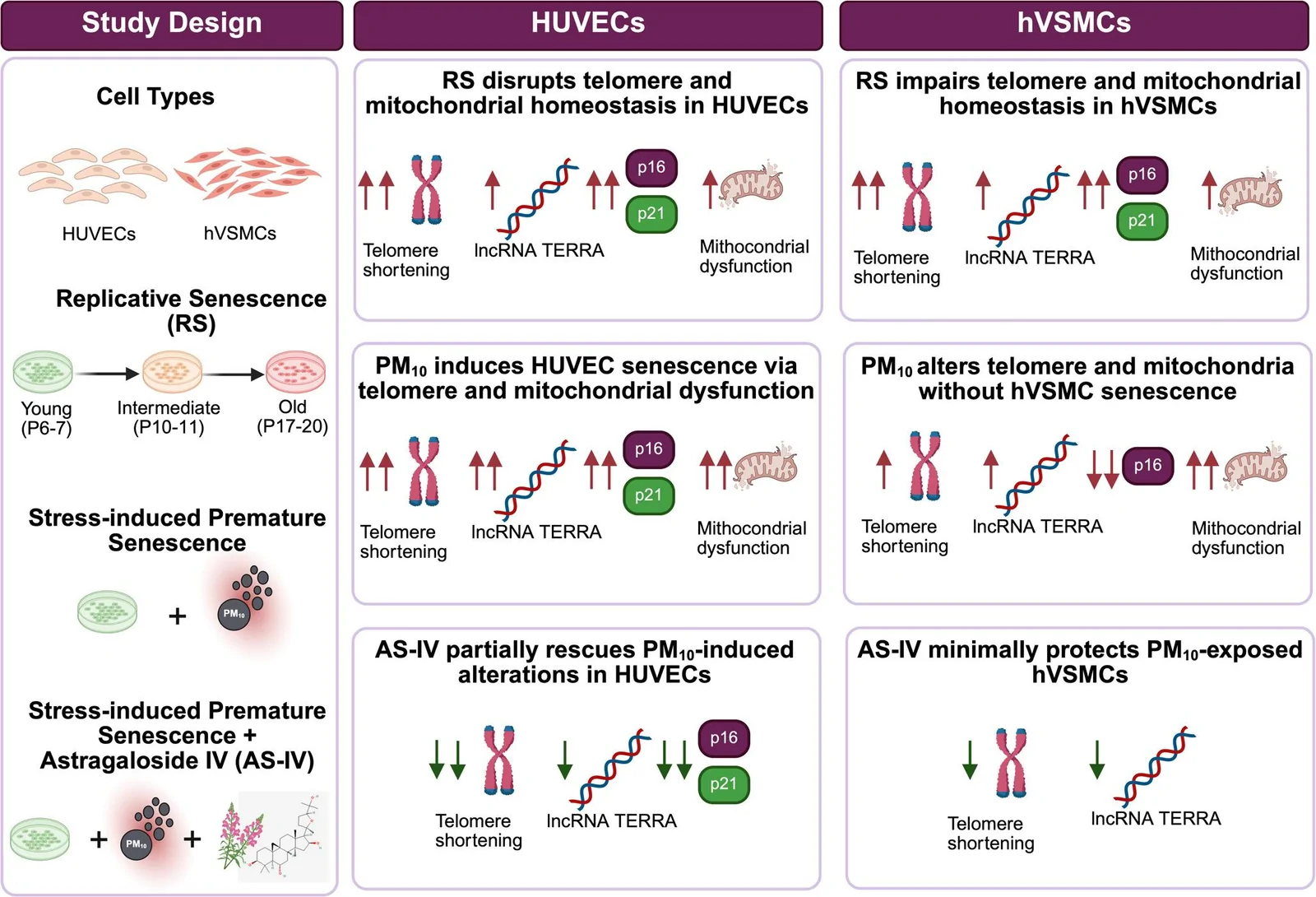

## Introduction

Cellular senescence in endothelial cells (ECs) and vascular smooth muscle cells (VSMCs) is recognized as a key contributor to vascular aging and to the onset and progression of atherosclerosis [1–4].

Senescent cells are characterized by a permanent growth arrest and a proinflammatory senescence-associated secretory phenotype (SASP). Senescence can arise through intrinsic mechanisms, such as replicative senescence (RS), or be triggered by extrinsic stressors, resulting in stress-induced premature senescence (SIPS). RS is primarily driven by progressive telomere shortening, leading to activation of the p53/p21^Cip1^ or p16^INK4a^/Rb pathways, whereas SIPS is induced by environmental or intracellular stress independently of replicative telomere attrition [5–7]. One important external trigger is air pollution, especially fine particulate matter (PM), such as PM_2.5 and_ PM_10_, which promote oxidative stress, cause DNA damage, and disrupt mitochondrial function, thereby accelerating vascular aging and atherosclerotic plaque formation, even in relatively young individuals or those without classical metabolic risk factors [8, 9].

Despite recent advances, the molecular mechanisms underlying vascular senescence remain incompletely understood, particularly across different vascular cell types and environmental contexts. Further research is needed to clarify these pathways, elucidate how external stressors, such as pollution, trigger premature senescence, and evaluate potential therapeutic strategies to slow vascular aging. Emerging evidence identifies telomere dysfunction and mitochondrial impairment as key drivers and interconnected hallmarks of cellular senescence rather than independent processes [10, 11]. Telomere dysfunction activates DNA damage responses, including p53-dependent pathways, which impair mitochondrial biogenesis and function, whereas mitochondrial-derived reactive oxygen species exacerbate telomeric DNA damage, further accelerating telomere dysfunction and senescence [10, 11]. Particulate matter may further amplify this cycle by simultaneously disrupting telomere integrity and mitochondrial homeostasis, thereby promoting premature vascular aging. However, the molecular mechanisms underlying these cell type-specific responses, particularly in the context of environmental pollution, remain incompletely understood.

Natural compounds that target these pathways have emerged as promising therapeutic candidates. Among them, *Astragalus membranaceus*, a traditional Chinese medicinal plant, has attracted considerable attention because of its telomerase-activating properties, ability to preserve telomere integrity, and potential to improve mitochondrial function [12, 13].

Accordingly, this study investigated the interplay between telomere dysfunction and mitochondrial impairment in human umbilical vein endothelial cells (HUVECs) and human coronary artery smooth muscle cells (hVSMCs) during both replicative and stress-induced senescence. Additionally, we evaluated the effects of exposure to traffic-related fine particulate matter using a road tunnel-derived particulate material representative of urban traffic emissions, alone and in combination with Astragaloside IV (AS-IV), on senescence-associated molecular and functional alterations. By comparing HUVECs and hVSMCs, we aim to clarify the mechanisms linking environmental pollution to vascular aging and to assess the therapeutic potential of AS-IV.

## Materials and Methods

### Chemicals and Reagents

Fine dust (ERMCZ-120), collected from the Wislostrada road tunnel (Warsaw, Poland), was purchased from Sigma-Aldrich (St. Louis, MO, USA). The ERMCZ-120 material is officially processed and classified as a PM_10_-like certified reference material consisting of particles with an aerodynamic diameter predominantly below 10 µm (PM_10_ fraction). Its composition reflects the complex mixture of traffic-derived particles, including inorganic components (metals and mineral elements), carbonaceous material, and other combustion-related constituents. A stock solution was prepared in dimethyl sulfoxide (DMSO) and diluted in culture medium to obtain working concentrations. Astragaloside IV (AS-IV, Cat. 74777, Sigma-Aldrich, St. Louis, MO, USA), the chemical constituent of Astragali radix, was dissolved in DMSO and diluted similarly. In all experiments, the final DMSO concentration did not exceed 0.1% (v/v).

### Cell Cultures

Primary human coronary artery smooth muscle cells (hVSMCs) and human umbilical vein endothelial cells (HUVECs) were obtained from Promocell (Heidelberg, Germany) and cultured according to the manufacturer’s instructions. Cells were seeded in T-75 flasks (5,000 cells/cm^2^) or six-well plates (1,800 cells/cm^2^), with medium replacement every two days, and passaged at 80–90% confluence. All experiments of SIPS were performed at passages 5–6.

Replicative senescence (RS) was induced by serial passaging and classified as young (P6-7), intermediate (P10-11), and old (P17-20). SIPS was induced in cells at passages 5–6 by exposure to FD (50 or 100 μg/mL) for 48 h. For combined treatments, cells were exposed to FD (100 μg/mL, 48 h) followed by AS-IV (50 µM, 24 h). Control cells received vehicle alone (DMSO).

### Senescence-Associated β-Galactosidase Staining

SA-β-Gal activity was assessed using the Cellular Senescence Assay Kit (Cat.KAA002; Sigma-Aldrich, St. Louis, MO, USA) according to the manufacturer’s instructions. Cells were incubated with staining solution at 37 °C overnight, and imaged using a Leica Mateo FL Digital Fluorescence Microscope. Quantification was performed with ImageJ software (v1.53c; National Institutes of Health, Bethesda, MD, USA).

### Mitochondrial and Nuclear Fluorescence Staining

At the end of the experimental protocol, cells were fixed by sequential incubation with 2% formaldehyde for 5 min, followed by 4% formaldehyde for 6 min. After fixation, cells were incubated with the mitochondrial fluorescent probe BioTracker 488 Green Mitochondria Dye (Cat. SCT136; Sigma-Aldrich, St. Louis, MO, USA) at a final concentration of 200 nM for 30 min at 37 °C to label mitochondria. Nuclear staining was performed using DAPI (4′,6-diamidino-2-phenylindole) (Cat. D3571, Invitrogen™, Thermo Fisher Scientific, Eugene, OR, USA). Fluorescence images were acquired using a Leica Mateo FL digital fluorescence microscope. For each experimental condition, 10 randomly selected fields were captured and analyzed using ImageJ software (version 1.53c; National Institutes of Health, Bethesda, MD, USA).

### Measurement of the Inner Mitochondrial Membrane Potential

Alterations in mitochondrial inner membrane potential (ΔΨm) were assessed using the mitochondria-targeted fluorescent probe JC-1 dye (5,5′,6,6′-tetrachloro-1,1′,3,3′-tetraethyl-imidacarbocyanine iodide) (Cat. T3168; Invitrogen™, Thermo Fisher Scientific, Eugene, OR, USA). JC-1 accumulates in mitochondria in a membrane potential-dependent manner, exhibiting a fluorescence emission shift from green (monomeric form) to red (J-aggregate form). A decrease in red fluorescence indicates mitochondrial depolarization, whereas an increase reflects mitochondrial hyperpolarization.

At the end of the experimental protocol, cells were incubated with JC-1 (100 nM) for 15 min at 37 °C in the dark, washed with PBS, and fluorescence images were acquired using a Leica Mateo FL digital fluorescence microscope. For each experimental condition, 10 randomly selected fields were captured. The ratio of red to green fluorescence intensity was quantified using ImageJ software (version 1.53c; National Institutes of Health, Bethesda, MD, USA) and used as an index of mitochondrial membrane potential (ΔΨm).

### DNA Extraction, Telomere Length, and Mitochondrial DNA Copy Number Analysis

Genomic DNA was extracted using the QIAamp DNA Mini Kit (Cat. 56304; Qiagen, Hilden, Germany). DNA purity and concentration were assessed by NanoDrop spectrophotometry (Thermo Scientific, Waltham, MA, USA), and samples with A260/A280 > 1.9 were used for analysis.

Telomere length (TL) and mitochondrial DNA copy number (mtDNA-CN) were quantified by qRT-PCR using a CFX384 Touch Real-Time PCR System (Bio-Rad, Hercules, CA, USA). Relative TL was calculated using the equation T/S ratio = 2^−ΔΔCt^, where Ct is a threshold cycle and ΔCt = Ct × telomere – Ct × single copy gene. mtDNA-CN was determined by amplifying the mitochondrial ND1 gene relative to the nuclear β-globin gene using the 2^−ΔCt^ method. All reactions were performed in triplicate. The primers used are reported in Table 1 (TEL and HBG for TL; and ND1 and B-GLOBIN for mtDNA-CN).

**Table 1:** Primer sequences used for quantitative PCR analysis

Gene	Sequence
p16^INK4a^	F 5′-CTTCCTGGACACGCTGGTG-3′ R 5′-GCATGGTTACTGCCTCTGGTG-3′
p21^Cip1^	F 5′-TGGAGACTCTCAGGGTCGAAA-3′ R 5′-GGCGTTTGGAGTGGTAGAAATC-3′
GAPDH	F 5′-TGGTATCGTGGAAGGACTCATG-3′ R 5′-GCTTCACCACCTTCTTGATGTC-3′
ND1	F 5′-AACATACCCATGGCCAAC-3′ R 5′-TCAGCGAAGGGTTGTAGTAGC-3′
B-GLOBIN	F 5′-GAAGAGCCAAGGACAGGTAC-3′ R 5′-CAACTTCATCCACGTTCACC-3′
TFAM	F 5′-GGCACAGGAAACCAGTTAGG-3′ R 5′-CAGAACACCGTGGCTTCTAC-3′
p53	F 5′-GAGCTGAATGAGGCCTTGGA-3′ R 5′-CTGAGTCAGGCCCTTCTGTCTT-3′
PGC-1α	F 5′-AGCCTCTTTGCCCAGATCTT-3′ R 5′-GGCAATCCGTCTTCATCCAC-3′
TRF2	F 5′-TTGTGGGGTCCTTGGACATA-3′ R 5′-CCAGTAGAAAACTGGTCAAGGAA-3′
HBG	F 5′- CGGCGGCGGGCGGCGCGGGCTGGGCGGCTTCATCCACGTTCACCTTG-3′ R 5′- GCCCGGCCCGCCGCGCCCGTCCCGCCGGAGGAGAAGTCTGCCGTT-3′
TEL	F 5′- TGTTAGGTATCCCTATCCCTATCCCTATCCCTATCCCTAACA R 5′- ACACTAAGGTTTGGGTTTGGGTTTGGGTTTGGGTTAGTGT
TRF1	F 5′-TGCTTTCAGTGGCTCTTCTG-3′ R 5′-ATGGAACCCAGCAACAAGAC-3′
TERRA	F 5′-GAATCCTGCGCACCGAGAT R 5′-CTGCACTTGAACCCTGCAATAC

### RNA Extraction and Real-Time PCR

Total RNA was isolated using the miRNeasy Mini Kit (Cat. 217004; Qiagen, Hilden, Germany) and quantified by NanoDrop spectrophotometry; samples with A260/A280 > 2.0 were used. cDNA was synthesized from 1 μg RNA using the iScript cDNA Synthesis Kit (Cat. 1708891; Bio-Rad, Hercules, CA, USA).

Gene expression analyses were performed by qRT-PCR using SsoAdvanced Universal SYBR Green Supermix (Cat. 1725274; Bio-Rad, Hercules, CA, USA) on a CFX384 system. The sequences of the primers are listed in Table 1. PCR conditions consisted of an initial denaturation at 95 °C for 5 min, followed by 40 cycles of 95 °C for 10 s, 58 °C for 20 s, and 72 °C for 10 s. Melting curve analysis confirmed specificity. Expression levels were normalized to GAPDH and analyzed using CFX Software (Bio-Rad, Hercules, CA, USA).

### Statistical Analysis

Data were analyzed using StatView software (v5.0.1). Data distribution met parametric assumptions. Results are presented as mean ± SEM, and p < 0.05 was considered statistically significant. Group comparisons were performed by one-way ANOVA followed by Fisher’s post hoc test. All experiments were conducted in triplicate and independently replicated four times.

## Results

### Morphology and SA-β-Gal Staining

**Fig. 1:**
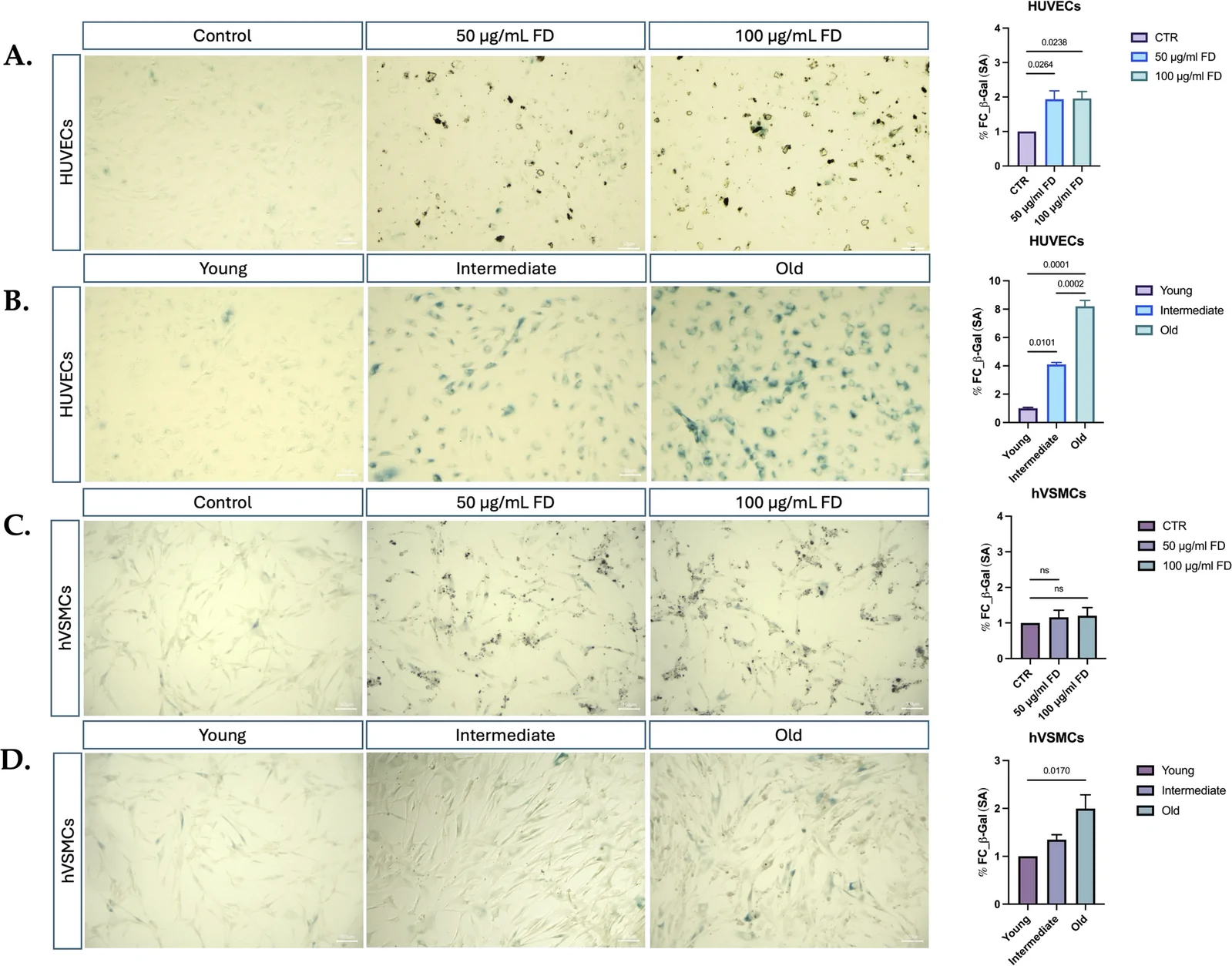
Representative images and quantification of SA-β-gal staining in HUVECs during (**A**) replicative senescence (Young, Intermediate, and Old) and (**B**) stress-induced premature senescence, (vehicle-treated control and cells treated with 50 and 100 µg/ml FD); Scale bars: 50 µm (**C**, **D**) Corresponding analysis for hVSMCs; Scale bars: 50 µm. Data are presented as the mean fold change (FC) from 4 independent experiments

During RS, both HUVECs and hVSMCs progressively developed a large, irregular morphology, accompanied by a rising proportion of SA-β-gal-positive cells (p = 0.0001 in HUVECs and p = 0.01 in hVSMCs) (Fig. 1). The percentage of SA-β-gal-positive cells rose progressively with passage number. Similar morphological changes and a significant increase in SA-β-gal positivity were also observed in HUVECs undergoing SIPS following exposure to both concentrations of FD for 48 h (p < 0.05 for both) (Fig. 1). In contrast, FD treatment did not significantly increase SA-β-gal positivity in hVSMCs, although non-significant changes in β-gal were observed (Fig. 1).

### Telomere Length and p16^INK4a^/p21^Cip1^ Expression in Replicative and Stress-Induced Senescence of HUVECs and hVSMCs

**Fig. 2:**
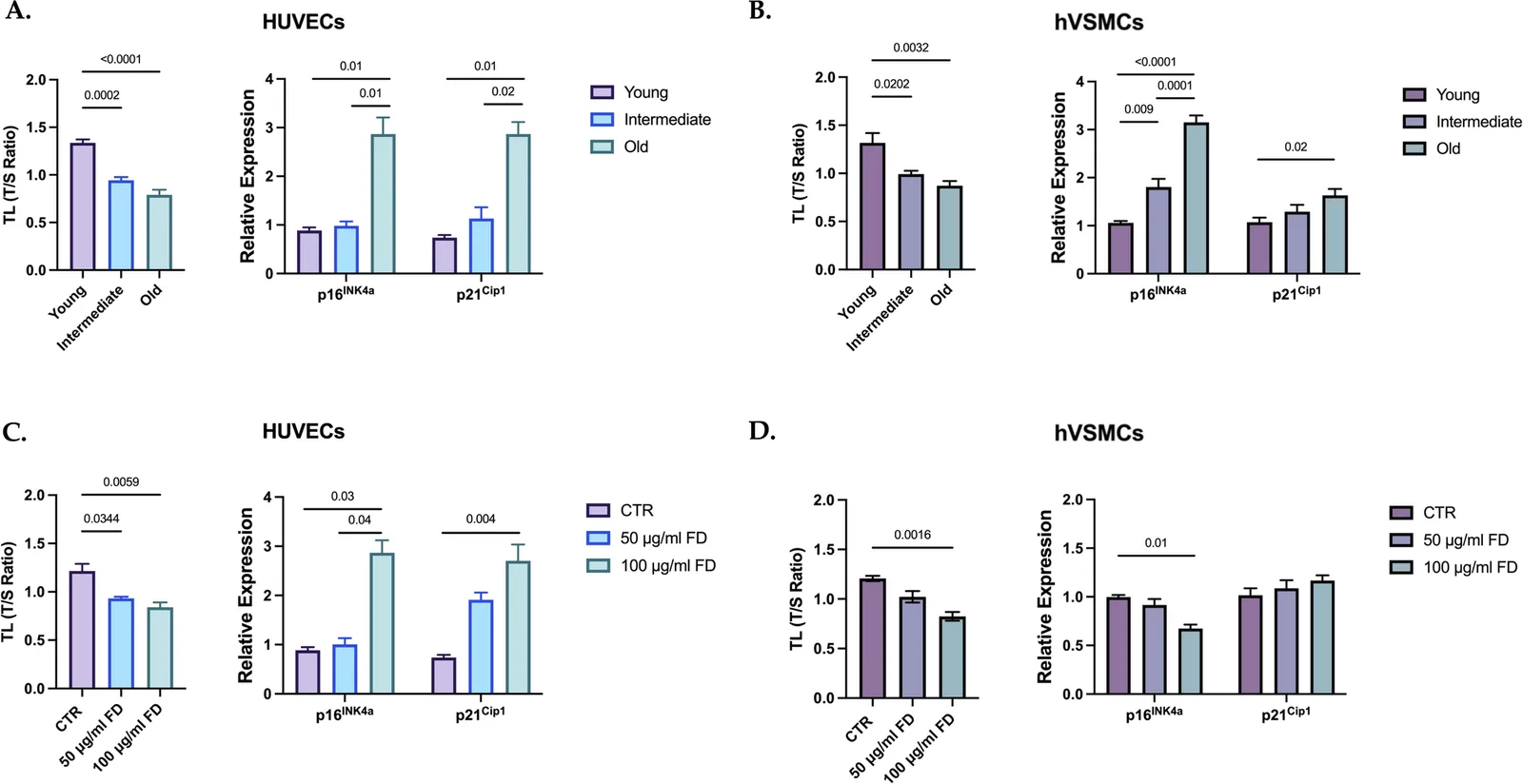
Telomere length as T/S ratio and relative expression of senescence markers in HUVECs during (**A**) RS and (**C**) SIPS, and in hVSMCs during (**B**) RS and (**D**) SIPS. Data are shown as relative expression (mean ± SEM) from 4 independent experiments

Compared with young cells, both old HUVECs (p < 0.0001) and old hVSMCs exhibited progressive telomere shortening (p = 0.003) and a transcriptional upregulation of the cell-cycle regulators p16^INK4a^ and p21^Cip1^ (p < 0.05 for all comparisons) (Fig. 2). Treatment with FD further reduced telomere length in both HUVECs and hVSMCs. In HUVECs, FD at 100 μg/ml significantly increased the expression of p16^INK4a^ and p21^Cip1^ compared with young cells (p = 0.005 for telomere length, p = 0.03 for p16^INK4a^, and p = 0.004 for p21^Cip1^) (Fig. 2). In hVSMCs, FD at 100 μg/ml also induced significant telomere shortening (p = 0.001), however, p16^INK4a^ expression was significantly reduced (p = 0.01), while no significant differences were observed for p21^Cip1^ expression (Fig. 2).

### TERRA and Shelterin Proteins in Replicative and Stress-Induced Vascular Senescence

**Fig. 3:**
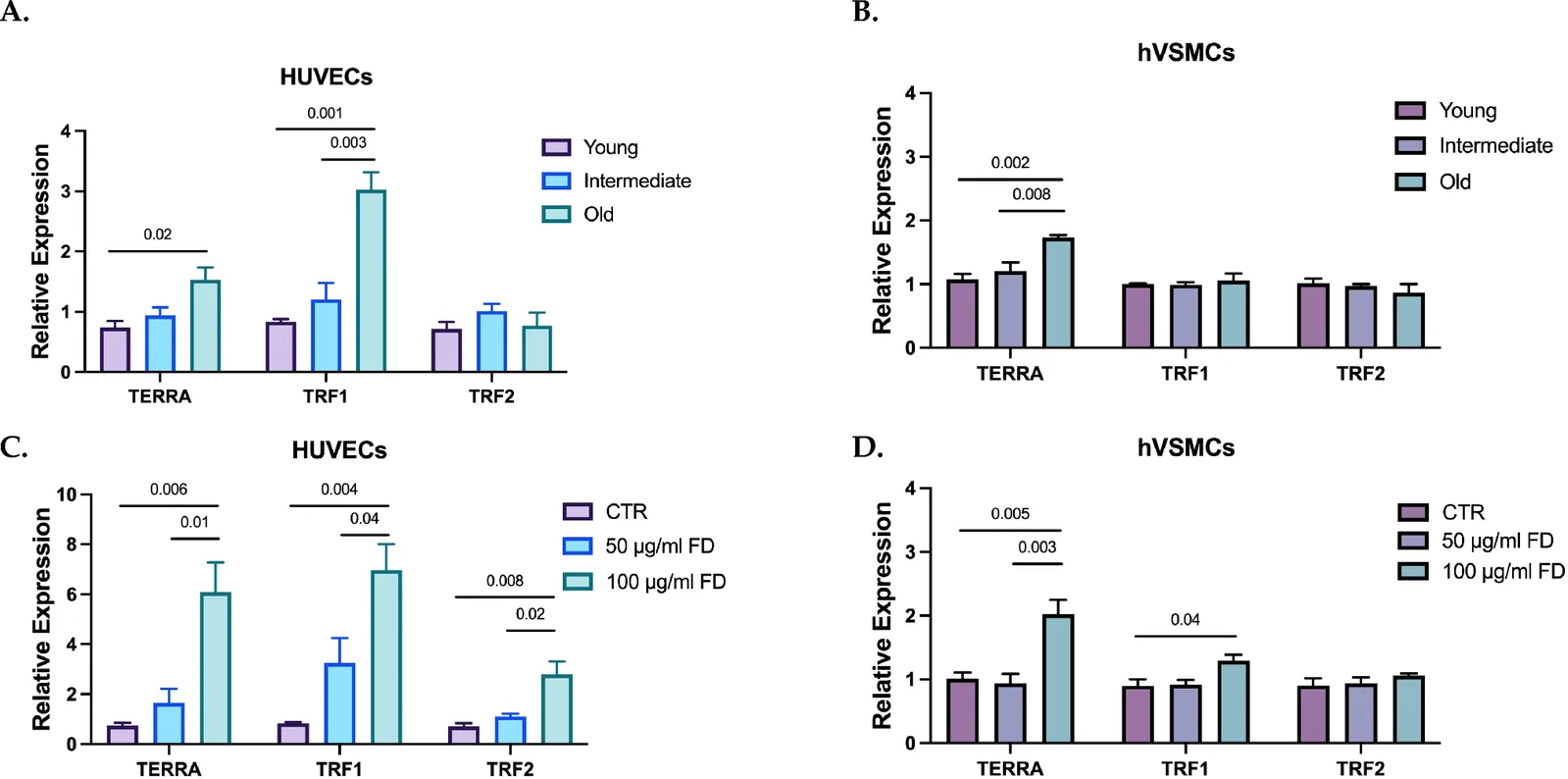
mRNA levels of the long non-coding RNA TERRA and shelterin components TRF1 and TRF2 were analyzed in HUVECs (**A**, **C**) and hVSMCs (**B**, **D**) under RS and SIPS conditions. Data are presented as mean ± SEM from 4 independent experiments

Given the essential roles of the long non-coding telomeric repeat-containing RNA (TERRA) and the shelterin proteins, particularly TRF1 and TRF2, in maintaining telomere integrity [14], we examined their expression under both RS and SIPS conditions. Both models showed a similar pattern (Fig. 3), characterized by increased TERRA levels in old cells and after high FD exposure in both HUVECs and hVSMCs (p < 0.05 for all comparisons), supporting a central role of TERRA in vascular telomere regulation. In the RS model, TRF1 expression was significantly increased only in intermediate and old HUVEC cells (p < 0.01 for all comparisons) (Fig. 3). In the SIPS model, a dose-dependent increase was observed for both TRF1 and TRF2 in HUVEC (p < 0.05 for all comparisons), whereas in hVSMCs a significant increase was detected only for TRF1 at the highest FD concentration (p = 0.04) (Fig. 3).

### Mitochondrial Biology in Replicative and Stress-Induced Vascular Senescence

**Fig. 4:**
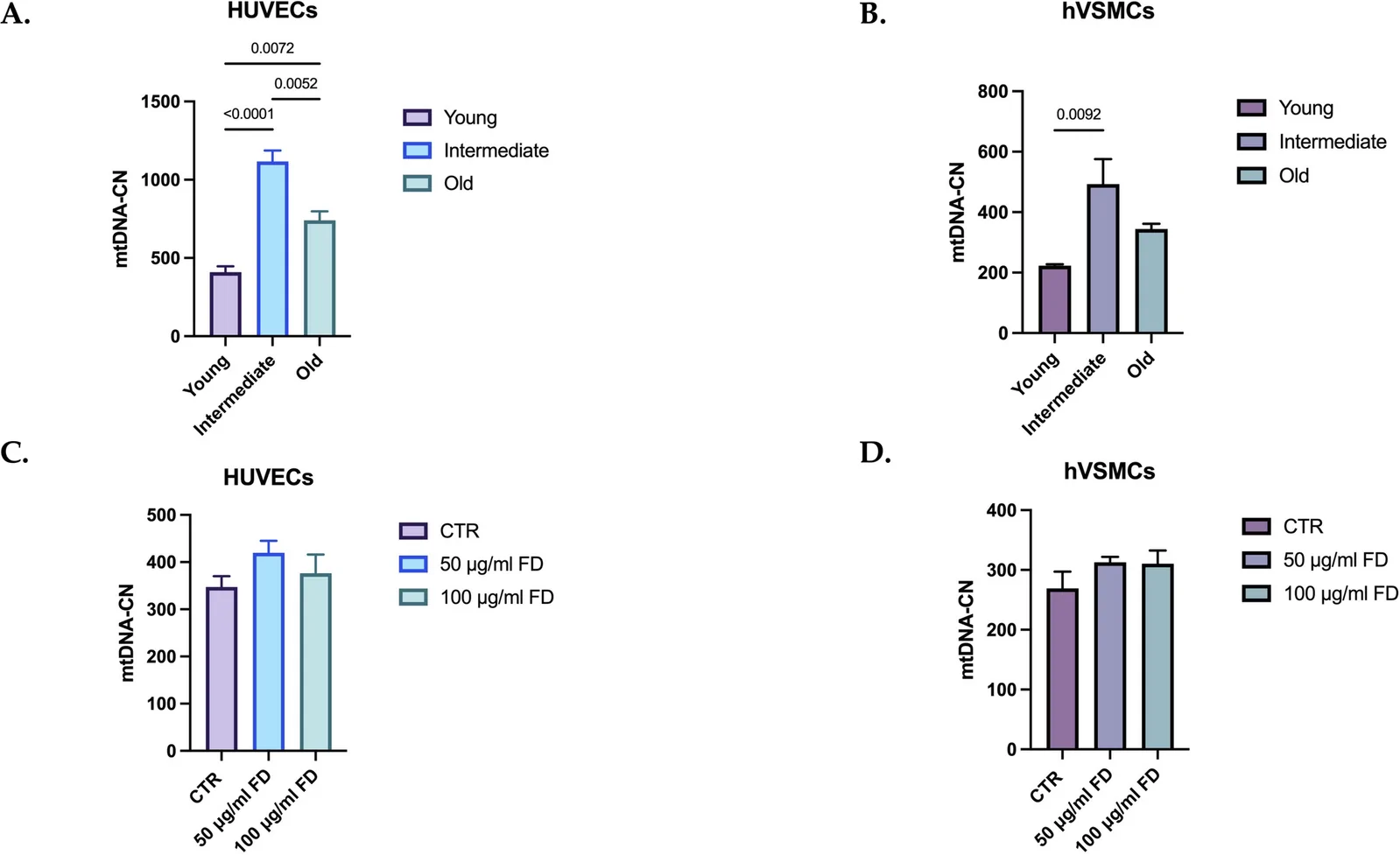
Quantification of mtDNA-CN in HUVECs (**A**, **C**) and hVSMCs (**B**, **D**) during RS and SIPS. Data are presented as mean ± SEM

To assess mitochondrial function, we first quantified mtDNA-CN in both RS and SIPS models. In the RS model, intermediate HUVECs (p < 0.0001) and intermediate hVSMCs (p = 0.009) showed an increase in mtDNA-CN compared with young cells, suggesting a compensatory response aimed at preserving mitochondrial function (Fig. 4). Old HUVECs also exhibited a significant increase relative to young cells (p = 0.007), but showed a significant decrease compared with intermediate cells (p = 0.005), whereas no significant differences were detected in old hVSMCs. In the SIPS model, a similar trend toward increased mtDNA-CN was observed in both HUVECs and hVSMCs, although the changes did not reach statistical significance (Fig. 4).

**Fig. 5:**
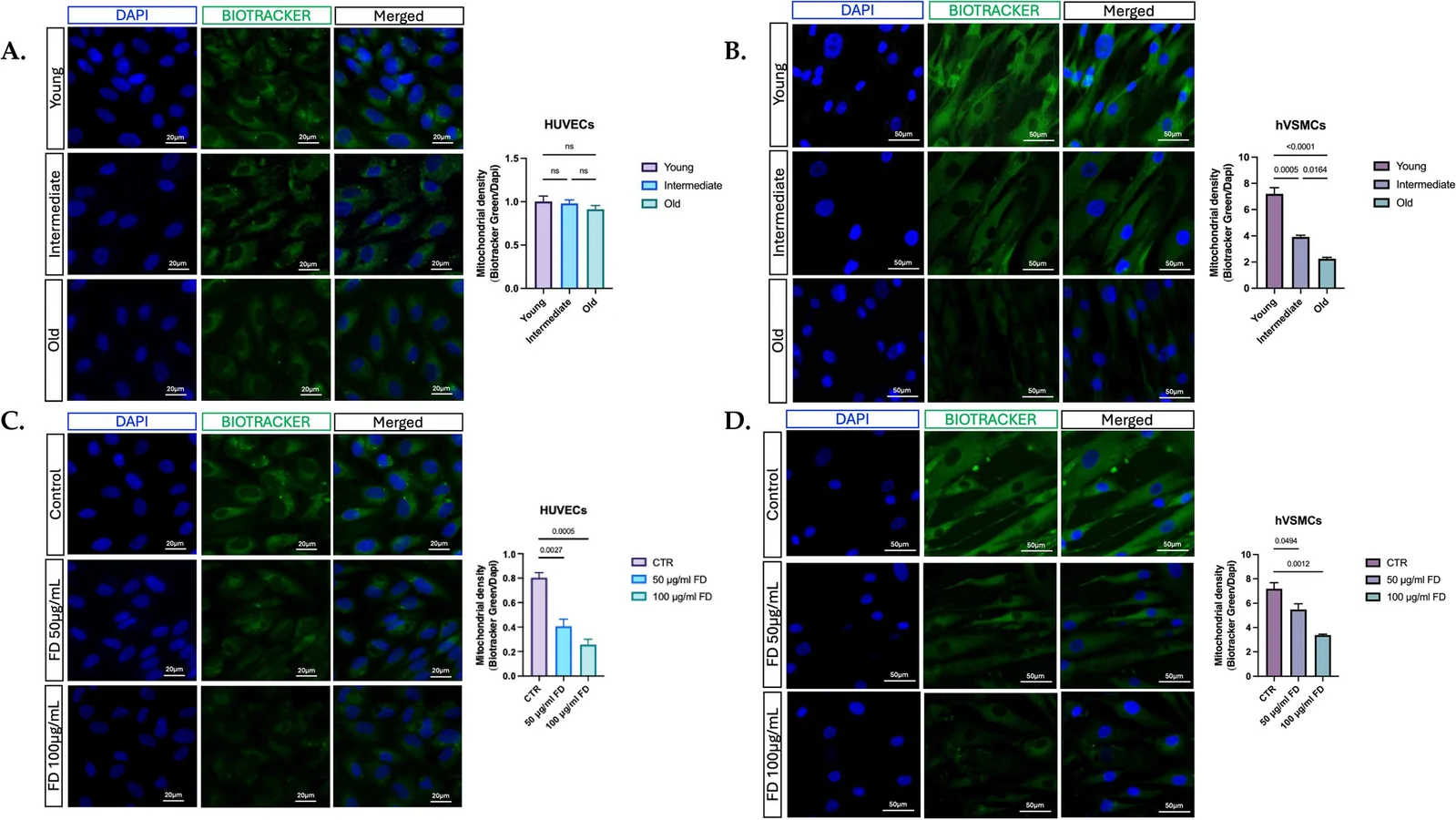
Representative fluorescence microscopy images of HUVECs stained with DAPI (blue) and BioTracker Green (green) to visualize nuclei and mitochondrial density, respectively (**A**, **C**). Scale bars: 20 µm. Corresponding analysis for hVSMCs (**B**, **D**). Scale bars: 50 µm. Data are presented as mean ± SEM from 4 independent experiments

**Fig. 6:**
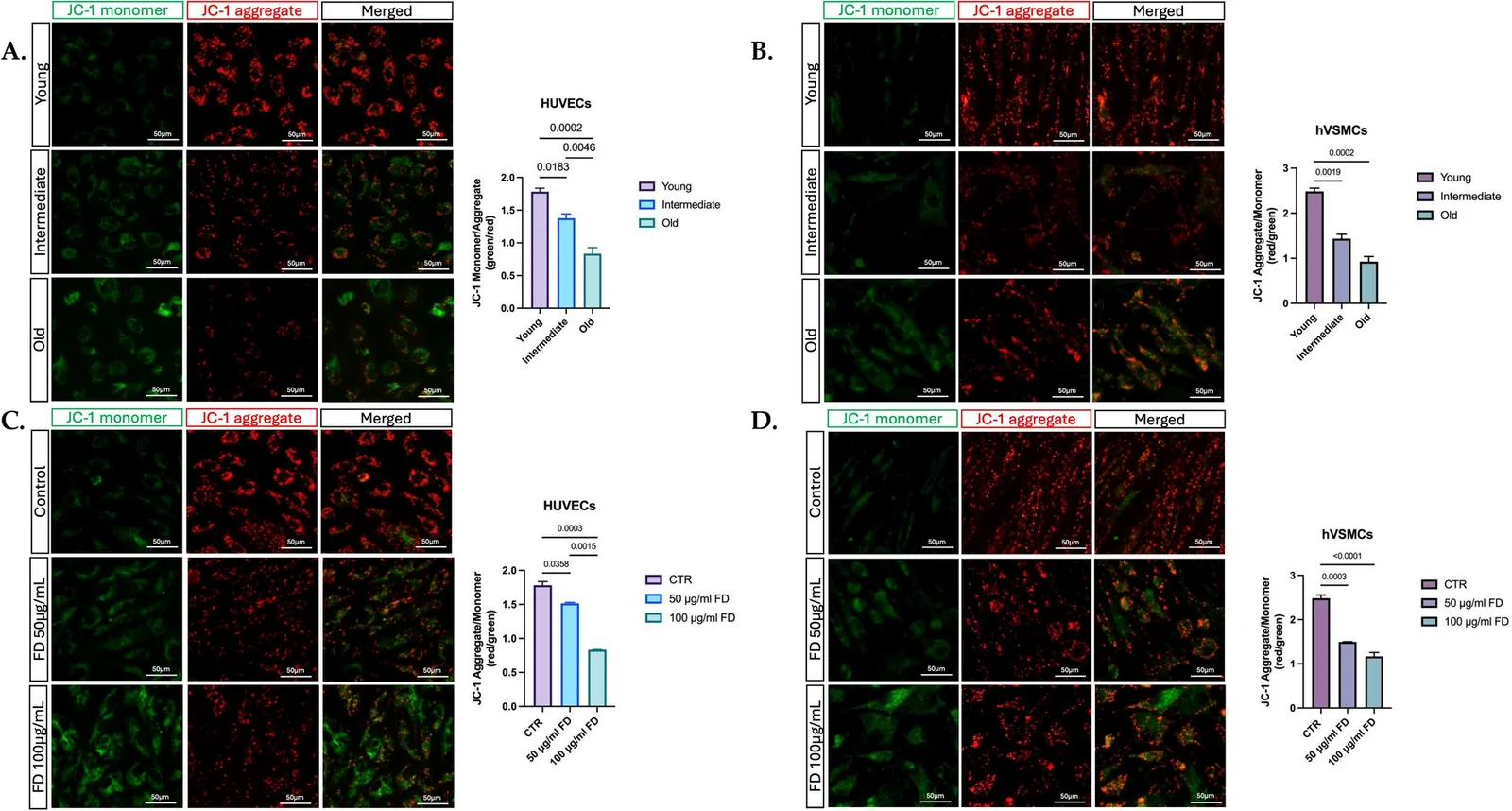
Representative images and JC-1 ratio quantification in HUVECs during (**A**) RS and (**C**) SIPS and in hVSMC during (**B**) RS and (**D**) SIPS. The shift from red (JC-1 aggregates) to green (JC-1 monomers) indicates mitochondrial depolarization. Scale bars: 50 µm. Data are presented as mean ± SEM from 4 independent experiments

Mitochondrial mass and mitochondrial membrane potential were assessed in both HUVECs and hVSMCs under RS and SIPS conditions. In the RS model, mitochondrial mass remained unchanged in HUVECs, whereas mitochondrial membrane potential was reduced in intermediate (p = 0.02) and old cells (p = 0.0002) compared with young cells, as indicated by a decreased red/green fluorescence ratio in JC-1 staining (Figs. 5 and 6). In hVSMCs, both mitochondrial mass and membrane potential were significantly decreased during RS in intermediate (p = 0.0005 for mass; p = 0.001 for potential) and old cells (p < 0.0001 for mass; p = 0.0002 for potential) compared with young cells. These parameters were also decreased following FD-treatment (p < 0.05 for all comparisons), highlighting a heightened vulnerability of these vascular cells to mitochondrial dysfunction. In the SIPS model, HUVECs also showed a significant reduction in both mitochondrial mass and activity (p < 0.05 for all comparisons), indicating a more pronounced impairment of mitochondrial function in both vascular cells (Figs. 5 and 6).

**Fig. 7:**
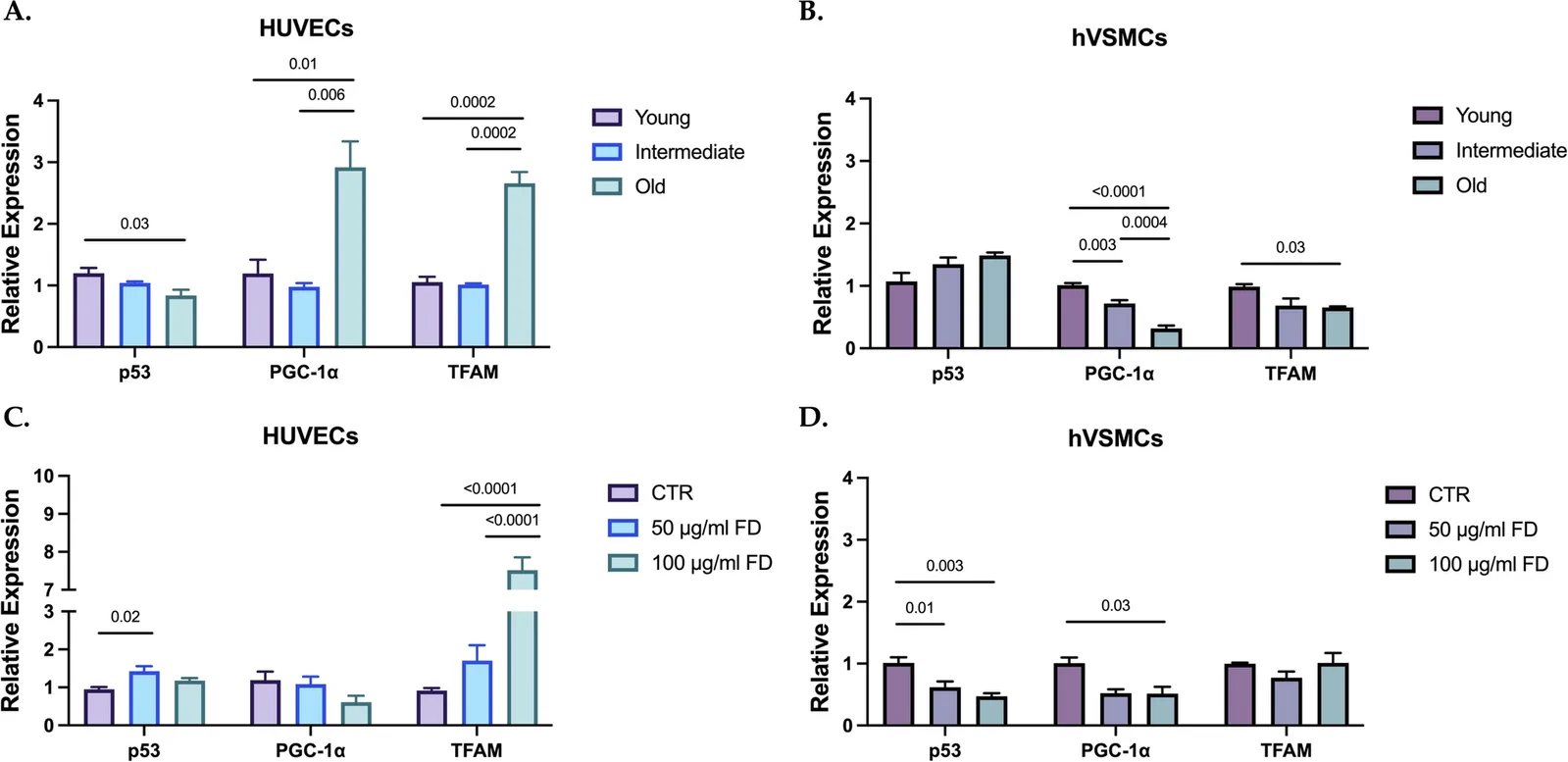
mRNA expression of genes involved in mitochondrial biogenesis and integrity in HUVECs (**A**, **C**) and hVSMCs (**B**, **D**) during RS and SIPS. Data are presented as mean ± SEM from 4 independent experiments

To further characterize mitochondrial function during senescence, we examined the expression of genes involved in mitochondrial biogenesis (PGC-1α, TFAM), as well as p53, a key regulator of mitochondrial quality control and genome integrity. In old HUVECs, p53 expression remained largely unchanged, whereas TFAM (p = 0.0002) and PGC-1α (p = 0.01) were significantly upregulated, possibly reflecting a compensatory response. In contrast, old hVSMCs showed a significant reduction in PGC-1α (p < 0.0001) and TFAM expression (p = 0.03) (Fig. 7). In the SIPS model, p53 expression was significantly increased in HUVECs at 50 μg/ml FD (p = 0.02), while TFAM expression was markedly upregulated at 100 μg/ml FD (p < 0.0001) (Fig. 7). In contrast, a significant dose-dependent decrease in p53 expression was observed in hVSMCs (p < 0.01), together with a reduction in PGC-1α expression at 100 μg/ml FD (p = 0.01) (Fig. 7).

### Combined Effects of FD and Astragaloside IV on Vascular Cells

**Fig. 8:**
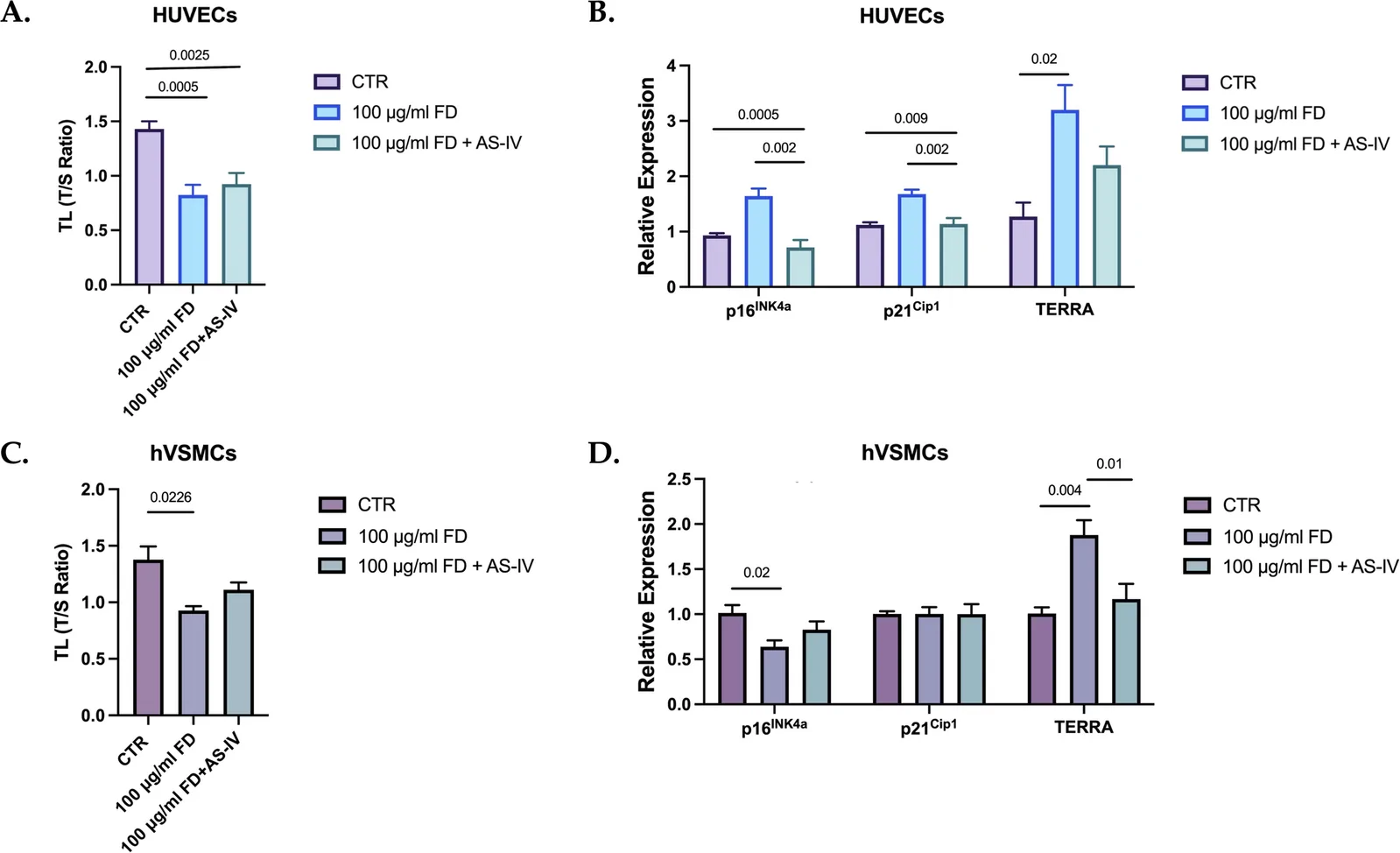
(**A**, **C**) Telomere length measured as T/S ratio in HUVECs and hVSMCs. (**B**, **D**) Relative mRNA expression of senescence markers and lncRNA TERRA in HUVECs and hVSMCs. Experimental groups include vehicle-treated control cells (CTR), 100 µg/ml FD, and combined treatment (FD+AS-IV). Data are presented as mean ± SEM from 4 independent experiments

To evaluate the potential protective effects of Astragalus membranaceus, we finally investigated the combined effects of FD at 100 μg/mL and AS-IV in HUVECs and hVSMCs. In HUVECs, the combined treatment resulted in a trend of increased TL when compared to cells treated with FD alone. The expression of p16^INK4a^ and p21^Cip1^ was significantly reduced by the combined treatment compared with both FD-treated and control cells (p < 0.05 for all comparisons) (Fig. 8). In hVSMCs, no significant differences were observed in TL or in the expression of cell-cycle regulators, although a trend toward increased TL was detected in the presence of AS-IV (Fig. 8).

**Fig. 9:**
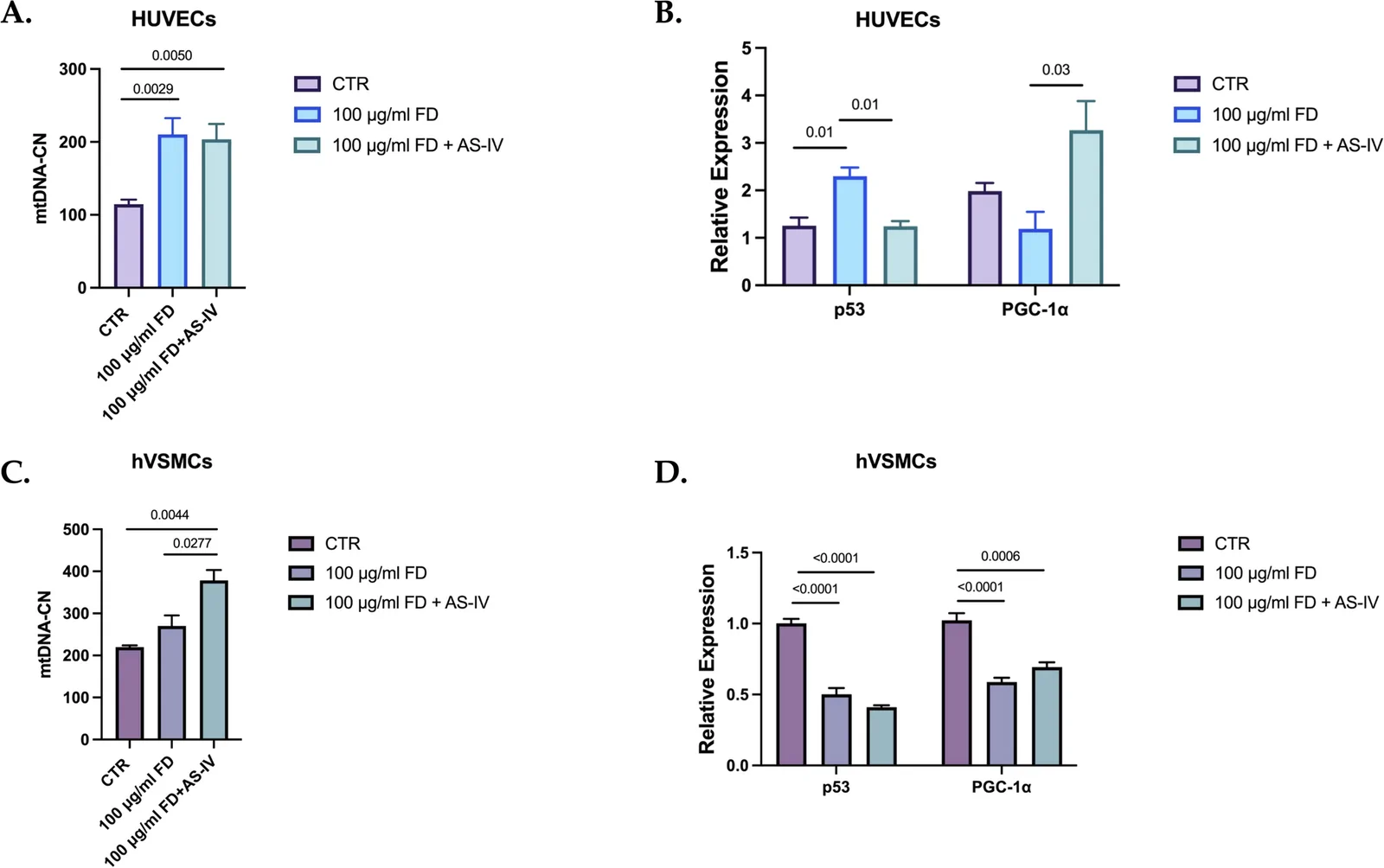
(**A**, **C**) mtDNA-CN and (**B**, **D**) relative expression of p53 and PGC-1α in HUVECs and hVSMCs treated with FD and AS-IV. Data are presented as mean ± SEM from 4 independent experiments

Additionally, AS-IV attenuated FD-induced TERRA upregulation in HUVECs and significantly reduced it in hVSMCs (p = 0.01), restoring them to control levels (Fig. 8). Regarding mitochondrial biology, mtDNA-CN increased following exposure alone in HUVECs (p = 0.002), as well as after combined FD and AS-IV treatment (p = 0.005). In contrast, in hVSMCs, higher mtDNA levels were observed only after AS-IV administration compared with both control (p = 0.004) and FD-only-conditions (p = 0.03), as shown in Fig. 9. In HUVECs, AS-IV reduced p53 expression relative to FD alone (p = 0.01), and was associated with increased PGC-1α expression (p = 0.03), suggesting partial restoration of mitochondrial regulatory pathways (Fig. 9). In contrast, in hVSMCs both p53 (p < 0.0001) and PGC-1α (p < 0.0001) remained downregulated under FD exposure and combined treatment, indicating persistent mitochondrial vulnerability despite AS-IV administration (Fig. 9).

## Discussion

In this study, we provide a comparative analysis of replicative and fine dust-induced premature senescence in HUVECs and hVSMCs, revealing distinct cell type-specific telomeric and mitochondrial responses to particulate matter exposure and the potential protective effects of astragaloside IV.

### Telomeric Biology During RS and SIPS in Vascular Cells

Although RS and SIPS share several molecular hallmarks, including telomere shortening, altered telomere-associated transcripts, and mitochondrial dysfunction [1–5], they represent distinct aging trajectories. RS reflects progressive, division-dependent telomeric shortening, whereas SIPS results from acute environmental stress that disrupts cellular homeostasis [3–7, 9].

In our study, both HUVECs and hVSMCs exhibited classical hallmarks of RS, including SA-β-gal positivity, telomere shortening, and upregulation of p16^INK4a^ and p21^Cip1^, supporting telomere attrition as a key driver of vascular aging [4, 15]. In contrast, SIPS revealed marked cell-type specificity. FD exposure rapidly induced a senescent phenotype in HUVECs, characterized by increased SA-β-gal activity, telomere shortening, and activation of p16^INK4a^/p21^Cip1^. Conversely, hVSMCs showed relative resistance to FD-induced senescence, despite telomere shortening, with limited activation of p16^INK4a^ expression and unchanged p21^Cip1^ levels.

A novel aspect of this study is the investigation of the long non-coding RNA TERRA, a key regulator of telomere biology involved in heterochromatin formation, telomerase regulation, and DNA damage responses at chromosome ends [15, 16]. HUVECs exhibited marked TERRA upregulation during both RS and SIPS, suggesting an early adaptive response to telomeric stress that may become insufficient under persistent environmental challenge, contributing to telomere instability and irreversible growth arrest. In contrast, hVSMCs displayed more gradual changes in TERRA and shelterin components during RS and minimal alterations following FD exposure, consistent with their greater resistance to SIPS.

These findings are consistent with previous studies showing that vascular replicative senescence, particularly in endothelial cells, is driven by progressive telomere shortening and activation of senescence pathways [17–19]. Loss of telomere protection promotes sustained DNA damage signaling, oxidative stress, inflammation, and permanent cell cycle arrest [17–19].

However, under conditions of stress-induced premature senescence, telomeres remain highly vulnerable to damage, and stressed VSMCs may respond through phenotypic transitions, including pro-inflammatory, osteogenic, or calcific remodeling, which contribute to atherosclerotic vascular disease [1, 20–23]. These responses likely reflect the intrinsic phenotypic plasticity of VSMCs, which, unlike endothelial cells, can adapt to environmental stress through dynamic and potentially reversible shifts between contractile, synthetic, inflammatory, and metabolic states rather than immediate activation of irreversible growth arrest [23]. Therefore, telomere dysfunction in hVSMCs may represent an early stress signal that promotes phenotypic remodeling rather than classical senescence, potentially contributing to vascular disease progression through altered cellular functions without full engagement of canonical senescence pathways.

### Mitochondrial Biology During RS and SIPS in Vascular Cells

During RS, both HUVECs and hVSMCs mitochondrial analyses revealed a biphasic and context-dependent response, with transient increases in mtDNA copy number during early replicative senescence likely reflecting compensatory mitochondrial biogenesis, followed by functional decline at older stages.

Notably, in HUVECs mitochondrial membrane potential declined without a concomitant reduction in mitochondrial mass, suggesting qualitative mitochondrial dysfunction rather than organelle loss [24]. Such impairment is a well-recognized feature of vascular aging and has been closely linked to oxidative stress, reduced bioenergetic efficiency, and the propagation of the senescent phenotype [24, 25]. Conversely, hVSMCs exhibited a decline in mitochondrial membrane potential accompanied by a reduction in mitochondrial mass during RS.

Compared with RS, FD-induced SIPS elicited a more acute impairment of mitochondria in HUVECs, characterized by concomitant reductions in both mitochondrial mass and activity, increased p53 expression, and induction of TFAM, suggesting mitochondrial failure as a key mediator of pollution-driven premature senescence. These findings are in line with evidence demonstrating that PM_2.5_ exposure promotes endothelial senescence through disruption of the SIRT1/PGC-1α/SIRT3 axis and inducing excessive accumulation of reactive oxygen species [26].

In hVSMCs, FD exposure reduced mitochondrial membrane potential and mitochondrial mass, as well as p53 and PGC-1α expression, further underscoring divergent mitochondrial stress responses between vascular cell types. Although VSMCs may be relatively resistant to classical senescence induction, they are known to be highly sensitive to a variety of stressors, including oxidative and metabolic stress. Mitochondrial alterations in these cells may promote vascular inflammation and phenotypic switching, contributing to disease progression through mechanisms distinct from irreversible growth arrest [20, 23, 27].

### Effects of Astragaloside IV on Vascular Senescence

AS-IV, a major bioactive saponin derived from *Astragalus membranaceus*, has attracted increasing interest due to its pleiotropic cardiovascular protective properties [13]. Although not currently approved as an independent pharmacological treatment, AS-IV has been extensively investigated in preclinical models, demonstrating beneficial effects on endothelial function, oxidative stress, inflammation, and vascular remodeling [13]. These effects are mediated through multiple stress-response pathways, including activation of the Nrf2 antioxidant pathway, inhibition of NF-κB-dependent inflammatory signaling, and regulation of mitochondrial homeostasis via AMPK/PGC-1α signaling [13]. Emerging evidence further suggests that AS-IV may mitigate telomere shortening and cellular senescence, supporting its potential as a therapeutic strategy for targeting vascular aging and atherosclerosis [13].

In our study, combined FD and AS-IV treatment partially restored telomere length, reduced p16^INK4a^ and p21^Cip1^ expression, attenuated TERRA upregulation, and improved mitochondrial regulatory pathways in HUVECs. In contrast, hVSMCs show a limited response to AS-IV. Although treatment effectively normalized TERRA levels and increased mtDNA copy number, persistent downregulation of p53 and PGC-1α following both FD and combined treatment suggests incomplete recovery of mitochondrial homeostasis and limited activation of protective pathways.

Our findings are consistent with previous studies demonstrating that AS-IV exerts multiple protective effects in vascular cells [13]. In HUVECs, AS-IV enhances viability and proliferation, while attenuating apoptosis, oxidative stress, and inflammatory responses. In VSMCs, it generally inhibits pathological proliferation and migration and promotes a contractile phenotype, potentially limiting adverse vascular remodeling [13].

### Strengths and Limitations

A key strength of this study is the comparative analysis of replicative and fine dust-induced premature senescence in two major vascular cell types. This approach demonstrates coordinated alterations in telomere length, TERRA expression, and mitochondrial regulatory pathways during vascular aging, and highlights the heightened susceptibility of endothelial cells to pollution-related stress. Importantly, the treatment of AS-IV enhances the translational relevance of these findings by suggesting that pollution-induced cellular stress may be, at least in part, reversible.

Nevertheless, several limitations should be acknowledged. First, although HUVECs and hVSMCs are well-established experimental models, they cannot fully recapitulate the complexity of aged or diseased vascular tissues in vivo, which are influenced by donor characteristics, chronic inflammatory and metabolic stress, and the vascular microenvironment. In addition, exposure to fine dust in vitro models represents an acute and simplified approximation of air pollution, which may not fully capture the effects of prolonged, low-level exposures experienced in real-world human populations. Second, mechanistic conclusions regarding the telomere-mitochondria crosstalk and the mode of action of AS-IV remain largely correlative, as key regulatory pathways were not directly manipulated.

Third, although mitochondrial homeostasis was assessed using complementary measures of mitochondrial DNA copy number, mitochondrial mass, and mitochondrial membrane potential, direct analyses of mitochondrial respiration and intracellular reactive oxygen species production were not performed. Likewise, although senescence was validated using established markers, including SA-β-gal activity, telomere shortening, and p16^INK4a^/p21^Cip1^ expression, additional characterization of the senescent phenotype was limited by the lack of assessment of key SASP factors, such as IL-6 and IL-8. The inclusion of these inflammatory mediators would have provided a more comprehensive evaluation of the senescent phenotype and its functional consequences. Finally, our findings are based on in vitro models, and future studies using in vivo models of particulate matter exposure will be essential to validate these mechanisms in a physiological context and to further assess the therapeutic potential of Astragaloside IV.

## Conclusion

Our findings reinforce the concept that vascular aging is driven by tightly interconnected telomeric and mitochondrial pathways, whose bidirectional crosstalk is a defining feature of both replicative and stress-induced senescence [10, 28]. Environmental stressors, especially air pollution, may intensify this crosstalk by simultaneously inducing oxidative stress, telomeric DNA damage, and mitochondrial dysfunction, thereby creating a feed-forward loop that promotes premature vascular aging. These results highlight the telomere-mitochondria axis as a promising therapeutic target and support the development of interventions designed to preserve telomere integrity and mitochondrial homeostasis to delay vascular senescence and reduce the cardiovascular consequences of long-term environmental exposure [10, 28].

Importantly, endothelial cells and vascular smooth muscle cells exhibited distinct responses to particulate matter exposure. While endothelial cells readily activated canonical senescence pathways, VSMCs showed limited induction of classical senescence markers, consistent with their greater phenotypic plasticity and ability to undergo adaptive remodeling rather than irreversible growth arrest [29–31]. This highlights the particular vulnerability of endothelial cells as early targets of pollution-induced vascular dysfunction.

Our findings are consistent with recent in vivo evidence showing that chronic PM_2.5_ exposure promotes telomerase inactivation, telomere shortening, and cellular senescence in multiple tissues, supporting telomere dysfunction as a key mechanism linking air pollution to accelerated biological and vascular aging [32].

Moreover, the protective effects of AS-IV, evidenced by preservation of telomere length, modulation of senescence markers, and partial restoration of mitochondrial regulatory pathways, suggest that targeting telomere- mitochondria crosstalk might represent a novel strategy to preserve endothelial function, particularly in high-risk, pollution-exposed populations.

Combined exposure experiments indicate that AS-IV modulates pollution-induced telomeric stress in a cell type-specific manner, attenuating telomere-associated stress signals such as TERRA upregulation and shelterin imbalance without reversing established senescence [12, 13, 33, 34]. This supports its role as a telomere-stabilizing, stress-buffering agent rather than a classical senescence-reversing compound, a distinction particularly relevant under chronic environmental stress.

This distinction is particularly relevant in the context of chronic environmental exposure, where preserving adaptive stress responses while limiting excessive telomeric dysfunction may be more physiologically advantageous than broadly suppressing senescence pathways.

From a clinical and public health perspective, our data further support air pollution as a potent accelerator of premature vascular aging-especially at the endothelial level-even in the absence of traditional cardiovascular risk factors. These findings highlight the need to integrate environmental risk assessment into cardiovascular prevention strategies and encourage further investigation of telomere-modulating interventions to delay vascular aging and mitigate pollution-induced vascular dysfunction.

## Data Availability

All data generated or analyzed during this study are included in this published article. Additional data are available from the corresponding author upon reasonable request.

## Abbreviations

AS-IV: Astragaloside IV
DAPI: 4′,6-Diamidino-2-Phenylindole
DMSO: Dimethyl Sulfoxide
ECs: Endothelial Cells
FD: Fine Dust
GAPDH: Glyceraldehyde 3-Phosphate Dehydrogenase
HUVECs: Human Umbilical Vein Endothelial Cells
hVSMCs: Human Coronary Artery Smooth Muscle Cells
JC-1: 5,5′,6,6′-Tetrachloro-1,1′,3,3′-tetraethyl-imidacarbocyanine iodide
mtDNA: Mitochondrial DNA
mtDNA-CN: Mitochondrial DNA Copy Number
PGC-1α: Peroxisome proliferator-activated receptor gamma coactivator 1-alpha
RS: Replicative Senescence
SASP: Senescence-Associated Secretory Phenotype
SIPS: Stress-Induced Premature Senescence
TL: Telomere Length
TERRA: Telomeric Repeat-Containing RNA
TRF1: Telomeric Repeat-Binding Factor 1
TRF2: Telomeric Repeat-Binding Factor 2
TFAM: Mitochondrial Transcription Factor A
ΔΨm: Mitochondrial Membrane Potential
SA-β-gal: Senescence-Associated β-Galactosidase
